# Downregulation of the NHE3-Binding PDZ-Adaptor Protein PDZK1 Expression during Cytokine-Induced Inflammation in Interleukin-10–Deficient Mice

**DOI:** 10.1371/journal.pone.0040657

**Published:** 2012-07-27

**Authors:** Henrike Lenzen, Maria Lünnemann, Andre Bleich, Michael P. Manns, Ursula Seidler, Anne Jörns

**Affiliations:** 1 Department of Gastroenterology, Hepatology and Endocrinology, Hannover Medical School, Hannover, Germany; 2 Institute of Laboratory Animal Science, Hannover Medical School, Hannover, Germany; 3 Institute of Clinical Biochemistry, Hannover Medical School, Hannover, Germany; 4 Center of Anatomy, Hannover Medical School, Hannover, Germany; University of Tokyo, Japan

## Abstract

**Background:**

Impaired salt and water absorption is an important feature in the pathogenesis of diarrhea in inflammatory bowel disease (IBD). We analyzed the expression of proinflammatory cytokines in the infiltrating immune cells and the function and expression of the Na^+^/H^+^ exchanger isoform 3 (NHE3) and its regulatory PDZ-adaptor proteins NHERF1, NHERF2, and PDZK1 in the colon of interleukin-10–deficient (IL-10^−/−^) mice.

**Methodology/Principal Findings:**

Gene and protein expression were analyzed by real-time reverse transcription polymerase chain reaction (*q*RT-PCR), *in situ* RT-PCR, and immunohistochemistry. NHE3 activity was measured fluorometrically in apical enterocytes within isolated colonic crypts. Mice developed chronic colitis characterized by a typical immune cell infiltration composed of T-lymphocytes and macrophages, with high levels of gene and protein expression of the proinflammatory cytokines interleukin-1β and tumor necrosis factor-α. In parallel, inducible nitric oxide synthase expression was increased while procaspase 3 expression was unaffected. Interferon-γ expression remained low. Although acid-activated NHE3 activity was significantly decreased, the inflammatory process did not affect its gene and protein expression or its abundance and localization in the apical membrane. However, expression of the PDZ-adaptor proteins NHERF2 and PDZK1 was downregulated. NHERF1 expression was unchanged. In a comparative analysis we observed the PDZK1 downregulation also in the DSS (dextran sulphate sodium) model of colitis.

**Conclusions/Significance:**

The impairment of the absorptive function of the inflamed colon in the IL-10^−/−^mouse, in spite of unaltered NHE3 expression and localization, is accompanied by the downregulation of the NHE3-regulatory PDZ adaptors NHERF2 and PDZK1. We propose that the downregulation of PDZ-adaptor proteins may be an important factor leading to NHE3 dysfunction and diarrhea in the course of the cytokine-mediated inflammatory process in these animal models of IBD.

## Introduction

Inflammatory bowel disease (IBD) is a cytokine-mediated chronic inflammatory disease [1] with diarrhea attributable to intestinal inflammation-mediated dysregulation in salt and water transport as well as leaky tight junctions. [2]. However, the pathogenesis of inflammatory diarrhea is only partially understood, and the proinflammatory cytokines and their role in inflammation-associated diarrhea are not known in detail. Different proinflammatory cytokines, in particular tumor necrosis factor (TNF)-α, interferon (IFN)-γ, and interleukin (IL)-1β, have been implicated as key mediators of inflammatory diarrhea in IBD [3], [4]. However, results have been inconsistent. A crucial contribution to the inflammatory disease process has been documented in particular for the proinflammatory cytokine TNF-α [5]–[7]. This is supported in particular by the observation of a significant improvement of IBD through anti–TNF-α antibody treatment in many patients [8], [9].

The action of a defined set of ion transport proteins dominates transmucosal ion and fluid movement [2], and the inhibition of electroneutral NaCl absorption plays a key role in the pathogenesis of inflammatory diarrhea [10]. In the intestinal tract, the Na^+^/H^+^ exchanger isoform 3 (NHE3) is the most important transport protein for Na^+^ and water absorption [11]. NHE3 is expressed in the whole intestine [12], and it exists in dynamic multiple large multiprotein complexes [13]. The regulation of NHE3 involves direct linkage to PDZ-domain–containing proteins, including NHERF1, NHERF2, and PDZK1 (NHERF3) members of the NHERF family [14]–[16]. These PDZ-domain–containing proteins are involved in a wide range of biological functions, including multiprotein complex formation, interaction with other transporters, and signal transduction [13].

The IL-10^−/−^ mouse model, which under conventional housing conditions spontaneously develops a generalized enterocolitis similar to human IBD [17], is an interesting animal model to characterize the relationships between cytokine-mediated immune responses and dysfunction of the intestinal mucosa. Using the IL-10^−/−^ mouse model and for comparison also the DSS (dextran sulphate sodium) model, we analyzed the expression of different proinflammatory cytokines as well as of the Na^+^ absorptive ion transporter NHE3 and the regulatory PDZ-adaptor proteins NHERF1, NHERF2, and PDZK1. When we found a strong alteration in the mRNA and protein expression of the PDZ-adaptor protein PDZK1, but not of NHE3, we wondered whether the normal NHE3 expression is accompanied by normal NHE3 function in this mouse model of IBD.

## Results

### Histopathological Alterations in the Inflamed Colonic Mucosa of IL-10^−/−^ Mice

H&E staining of the inflamed colon of IL-10^−/−^ mice showed clear signs of inflammation, characterized by an increase in mononuclear cells, infiltrating all layers of the mucosa with the highest accumulation in the submucosal layer. The mucosal layer in the inflamed colon analyzed in this chronic stage was intact but hyperplastic. In the colon of WT mice, mononuclear cells were only occasionally observed, mostly in the mucosal layer. The appearance of H&E sections of the colon from IL-10^−/−^ mice kept under SPF conditions did not differ from those of the WT controls (H&E sections not shown). A histopathological scoring yielded for colon from control WT and IL-10^−/−^ SPF mice scores of 0 and 0.3±0.3, respectively, while the score was 3.1±0.1 in the colon of IL-10^−/−^ mice under conventional housing conditions (n = 4 mice in each group).

### Pattern of Proinflammatory Cytokines and iNOS Gene Expression in IL-10^−/−^ Mice

To further assess the chronic inflammatory state in the colon of IL-10^−/−^ mice, we measured mRNA expression profiles by *q*RT-PCR of different proinflammatory cytokines and cell death markers. IL-1β and TNF-α were more than 10 times and 5 times, respectively, increased in the inflamed colonic mucosa of IL-10^−/−^ mice as compared to the non-inflamed colonic mucosa of WT mice (Table 1). In contrast, IFN-γ gene expression was very low in the colonic mucosa of WT controls and was not significantly increased in the inflamed colonic mucosa of IL-10^−/−^ mice (Table 1). The iNOS gene expression was significantly induced in the inflamed colonic mucosa of IL-10^−/−^ mice (Table 1). Procaspase 3 gene expression was not increased in the cells in the colonic mucosa. In the colon of IL-10^−/−^ SPF mice IL-1ß and TNF-α gene expression were not increased in comparison to WT mice. Also, IFN-γ gene expression was very low in the colonic mucosa of IL-10^−/−^ SPF mice like in WT mice. iNOS and procaspase 3 gene expression were also not affected in the IL-10^−/−^ SPF mice (Table S1).

**Table:** **Table 1.** Gene expression profile of the proinflammatory cytokines IL-1β, TNF-α, IFN-γ and iNOS and the cell death marker procaspase 3 in the colonic mucosa of WT and IL-10^−/−^ mice.

Gene	WT control	IL-10^−/−^	Fold change against WT control
IL-1β	3.4±1.0	34.6±5.1*	10.2
TNF-α	1.3±0.3	7.9±2.1*	6.1
IFN-γ	0.007±0.0	0.008±0.01	1.2
iNOS	4.7±3.3	71.9±22.8*	15.3
Procaspase 3	36.9±12.6	24.9±12.4	0.7

Results are expressed as the mean normalized expression and fold change of IL-10^−/−^ mice against WT controls. mRNA was quantified in relation to β-actin. Data are mean values ± SEM (from 5–6 experiments in each group).

* p<0.05 versus control.

### *In situ* RT-PCR Measurements of Proinflammatory Cytokine and iNOS Gene Expression

**Figure 1:**
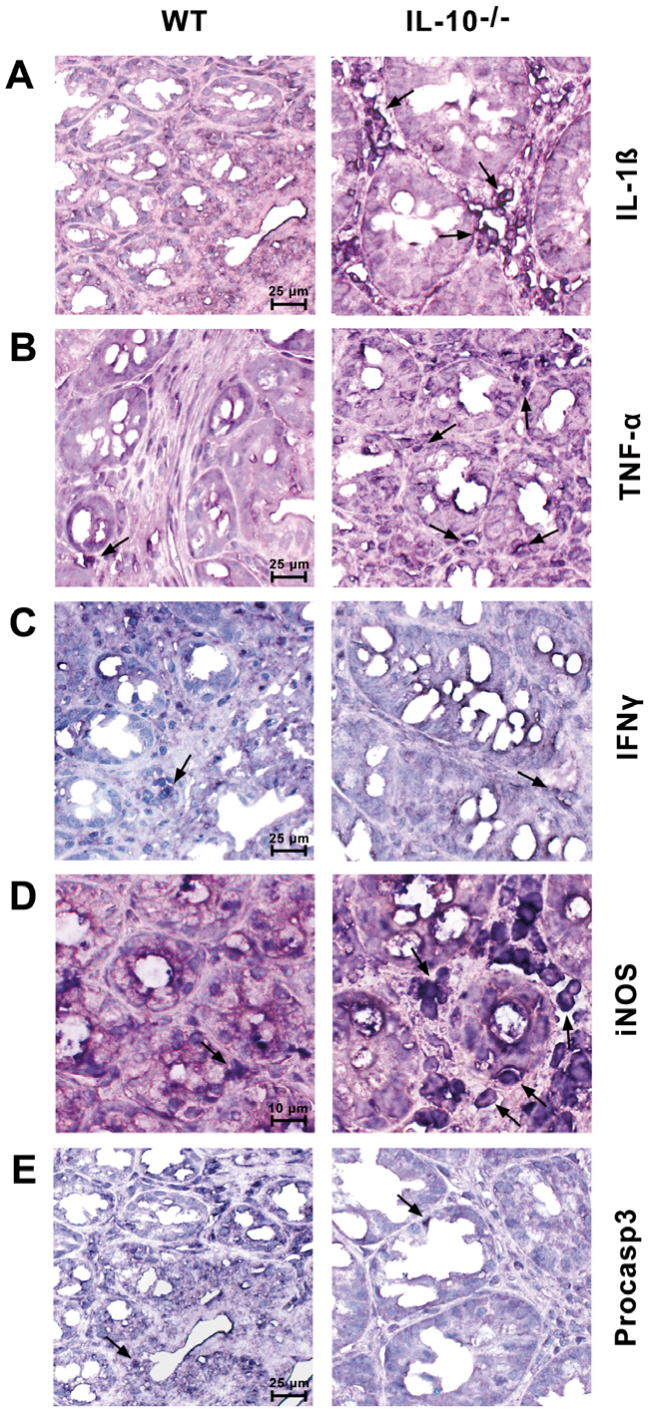
Gene expression by *in situ* RT-PCR of proinflammatory cytokines, iNOS, and procaspase 3 in the colonic mucosa. (A) and (B) High expression levels of IL-1β and TNF-α in infiltrating immune cells of IL-10^−/−^ mice. (C) IFN-γ exhibited scant expression in only single immune cells of IL-10^−/−^ mice and healthy WT mice. (D) High expression of iNOS in the immune cells surrounding the epithelial cells and in single immune cells in the connective tissue of the mucosa compared to WT mice. (E) Comparable procaspase 3 expression in WT and IL-10^−/−^ mice. Arrows show representative mononuclear cells. Representative images of three independent experiments.

To specify the cell types that produced the proinflammatory cytokines and induced iNOS, we performed *in situ* RT-PCR experiments. We identified a very high gene expression for the proinflammatory cytokines IL-1ß (Figure 1A) and TNF-α (Figure 1B) in the infiltrating immune cells in the mucosal (Figure 1) and submucosal (not shown) layers of the inflamed colonic mucosa of IL-10^−/−^ mice. No mRNA expression was observed in any other cell type of the colon, in particular not in enterocytes, goblet cells, muscle, or connective tissue. Only single immune cells with cytokine positivity were observed occasionally in the different layers of the colon from WT mice (Figure 1A and 1B). Faint mRNA expression of IFN-γ was detected only in a very few immune cells in the inflamed mucosa of IL-10^−/−^ mice and in the mucosa of WT mice (Figure 1C). The mRNA transcripts of the IL-1β–inducible enzyme iNOS, as measured by *in situ* RT-PCR, were found at very high expression levels only in infiltrating immune cells in the mucosal (Figure 1D) and submucosal (not shown) layers in the inflamed colon of IL-10^−/−^ mice. Interestingly, the iNOS-expressing immune cells were located close to the epithelial layer. Only faint mRNA expression for iNOS was observed occasionally in the immune cells of the colon under control conditions (Figure 1D). Faint procaspase 3 gene expression was observed occasionally in enterocytes as well as in infiltrating immune cells in healthy WT mice without any indication of an increase in these cell types in IL-10^−/−^ samples (Figure 1E).

### Protein Expression of Proinflammatory Cytokines by Double Immunofluorescence

**Figure 2:**
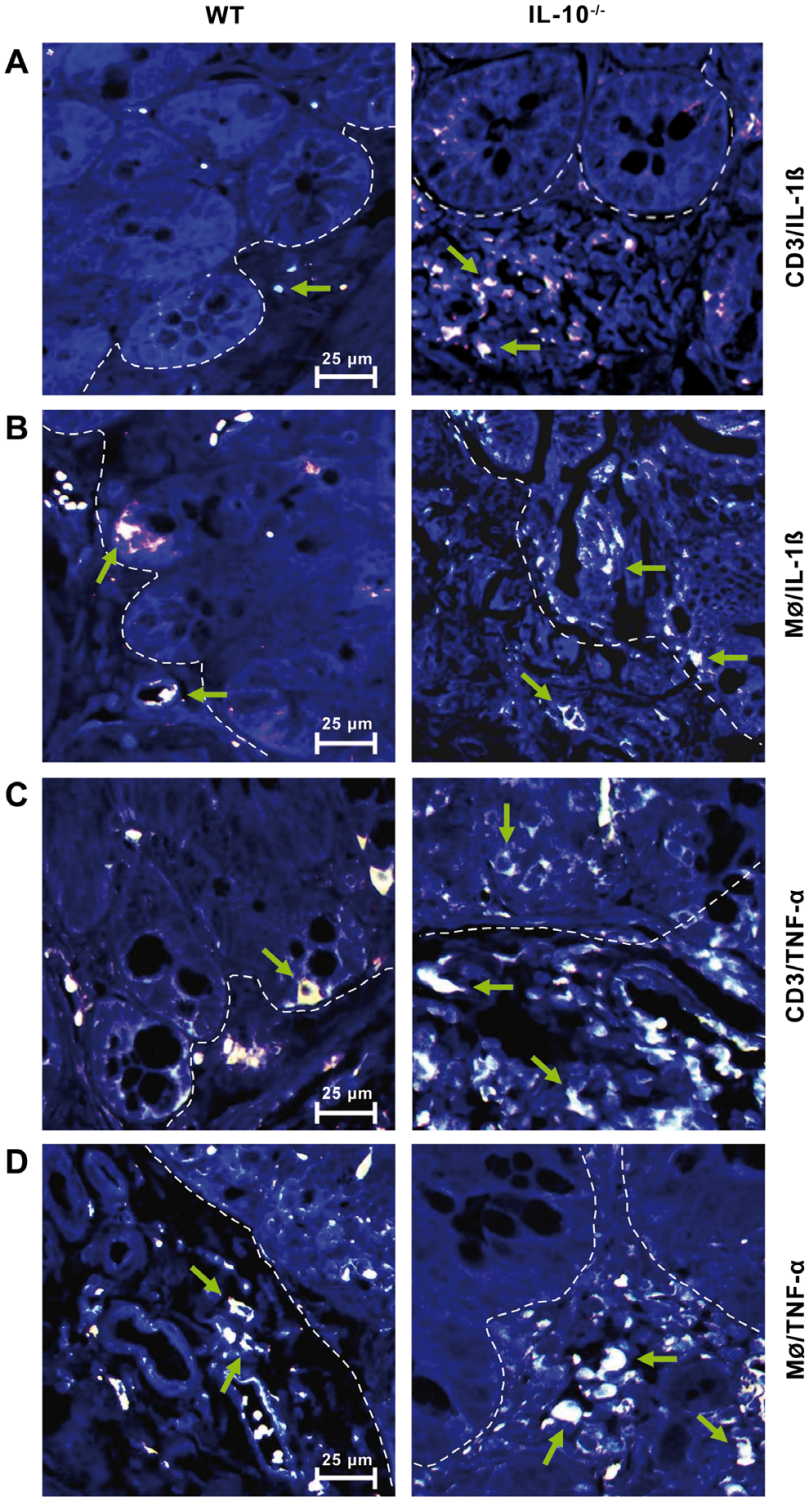
Double immunofluorescence staining of the proinflammatory cytokines IL-1β and TNF-α in infiltrating CD3 T-lymphocytes and macrophages. IL-10^−/−^ mice showed signs of inflammation with strong accumulation of CD3 T-lymphocytes and macrophages (Mø) in the mucosal and submucosal layer in the colon with high expression of the proinflammatory cytokines IL-1β and TNF-α. Only single-cytokine–positive CD3 T-lymphocytes and macrophages were observed in the layers of the colon from WT mice. The dashed line separates the epithelial layer from the connective tissue of the submucosal layer. Double immunofluorescence staining presents as mostly white in the overlay (instead of yellow) as an amalgamation of green and red due to the background staining with DAPI (blue). Representative cells are marked with arrows. Shown are representative images of three independent experiments.

To characterize the immune cell subtypes that express the cytokines also at the protein level, we used double immunofluorescence staining. The colonic mucosa of IL-10^−/−^ mice showed signs of inflammation with strong accumulation of IL-1β–positive (Figure 2A and 2B) and TNF-α–positive (Figure 2C and 2D) CD3 T-lymphocytes and macrophages in the mucosal and submucosal layers, whereas CD3 T-lymphocyte infiltration was more pronounced than the macrophage infiltration in comparison to healthy WT mice (Figure 2A and C versus Figure 2B and D). No protein expression was observed in enterocytes or goblet cells or in muscle and connective tissue (Figure 2). Single immune cells with cytokine positivity were observed occasionally in the different layers of the colon from WT controls.

### Gene Expression of NHE3 and PDZ-adaptor Proteins

**Figure 3:**
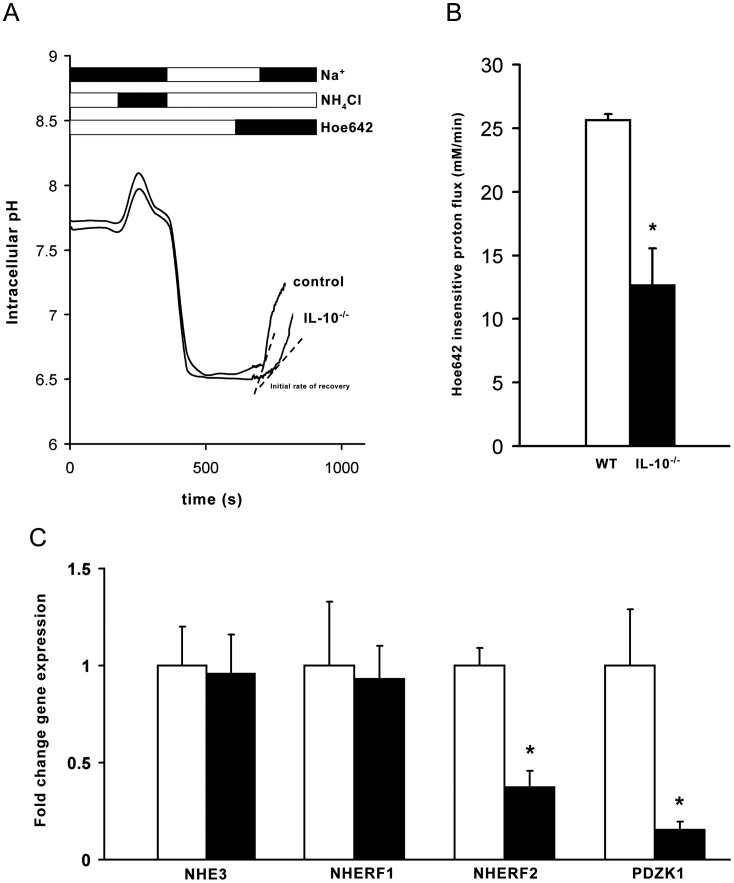
Fluorometric assessment of NHE3 activation by acid in colonic enterocytes in IL-10^−/−^ mice. (A) Exemplary pH curve. (B) Reduced NHE3 activity as measured by acid-activated Hoe642-insensitive proton flux in colonic enterocytes in IL-10^−/−^ mice (black columns) compared to healthy WT mice (open columns). n = 3 pairs of mice; * p<0.05 versus control. (C) Unaltered NHE3 gene expression but downregulation of the PDZ-adaptor proteins NHERF2 and PDZK1 in IL-10^−/−^ mice. The expression of NHERF1 was not affected. Results are expressed as a comparison of fold change in expression of NHE3, NHERF1, NHERF2 and PDZK1 in the inflamed and noninflamed colon (from 5–6 experiments in each group). mRNA was quantified in relation to ß-actin. *p<0.05 versus control.

Since the mice developed a pasty stool which is a sign of murine diarrheal disease, we measured the expression of NHE3, the dominant salt-absorptive transporter in the proximal part of the colon in IL-10^−/−^ mice. NHE3 gene expression by *q*RT-PCR was not affected by the inflammatory process in the colonic mucosa of IL-10^−/−^ mice (Figure 3C). Because of the reduced NHE3 activity despite normal gene expression, we further studied expression levels of the PDZ-adaptor proteins of the NHERF family, which play an important role for acid-activated NHE3 activity in the murine colon [18], [19]. We found that the gene expression of the two PDZ-adaptor proteins NHERF2 and PDZK1 was significantly reduced in the inflamed colonic mucosa of IL-10^−/−^ mice (Figure 3C) while, on the other hand, the inflammation did not affect NHERF1 expression. In the colon of IL-10^−/−^ SPF mice gene expression of NHE3 and the PDZ-adaptor proteins of the NHERF family did not differ from WT mice (Table S1).

### Unaltered NHE3 and Downregulation of NHERF2 and PDZK1 Gene Expression

**Figure 4:**
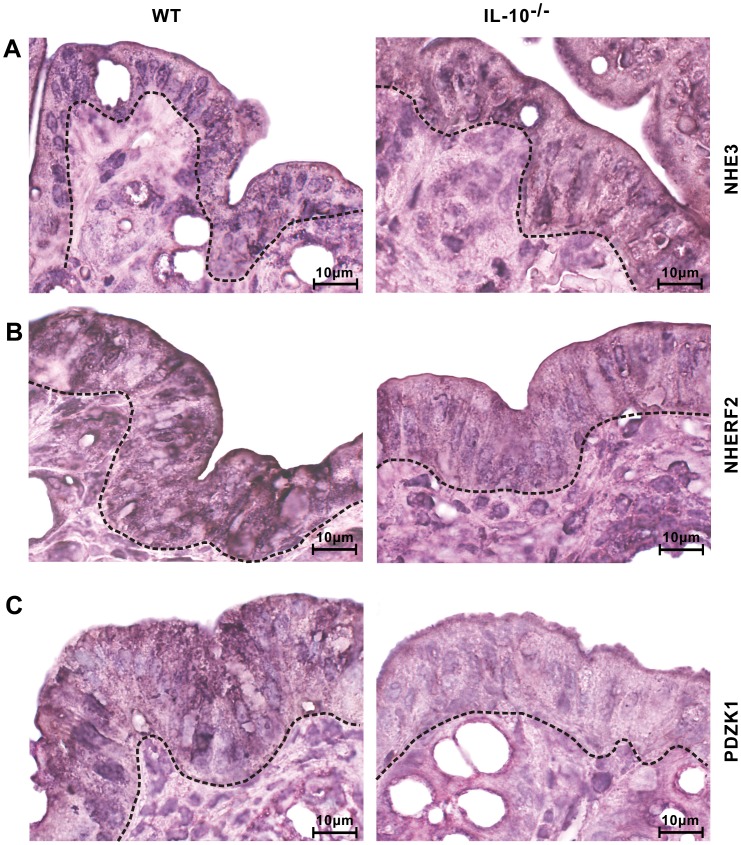
Gene expression by *in situ* RT-PCR of NHE3, NHERF2, and PDZK1 in the colonic mucosa. (A) Unaltered NHE3 expression levels in enterocytes of the epithelial layer of WT and IL-10^−/−^ mice. (B) mRNA expression of NHERF2 and (C) PDZK1 in the epithelial enterocytes were markedly decreased in IL-10^−/−^ mice. The dashed line separates the epithelial layer from the connective tissue of the lamina propria of the mucosa. Representative images of two independent experiments.

*In situ* RT-PCR was performed to further assess mRNA expression levels of NHE3 and the PDZ-adaptor proteins NHERF1, NHERF2, and PDZK1 in the epithelial cells of the colonic mucosa. Gene expression of NHE3 (Figure 4A) was localized only in the cytoplasm of enterocytes of the epithelial layer and showed no differences in expression between enterocytes of IL-10^−/−^ and WT mice. The gene expression of NHERF2 (Figure 4B) and PDZK1 (Figure 4C) was reduced in enterocytes of IL-10^−/−^ mice in comparison to WT controls. NHERF1 gene expression did not differ in enterocytes of the epithelial layer in IL-10^−/−^ and WT mice (data not shown). No mRNA expression for NHE3, NHERF1, NHERF2, or PDZK1 was observed in any cell type other than enterocytes in the colon.

### Protein Expression by Double Immunofluorescence of NHE3, NHERF2 and PDZK1

**Figure 5:**
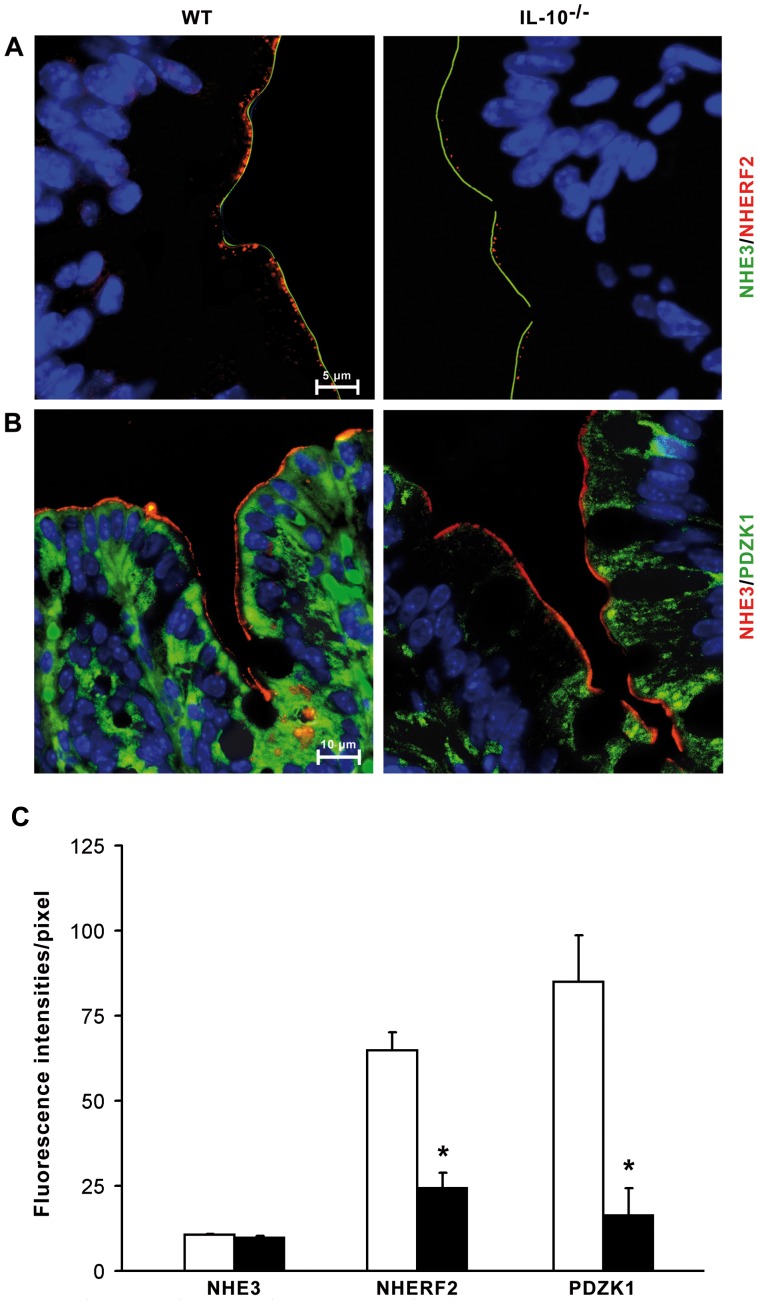
Double immunofluorescence staining and densitometric quantification of NHE3, NHERF2, and PDZK1 in enterocytes of the inflamed colon. (A) and (B) NHE3 protein expression was clearly restricted to the apical region of the enterocytes in colonic mucosa of diseased IL-10^−/−^ mice and WT mice in comparable intensities. (A) NHERF2 protein expression (red) was restricted to the apical region of the enterocytes in a dotted-like fashion in association with the NHE3 staining (green). The number of red dots was markedly reduced in diseased IL-10^−/−^ mice. (B) The PDZK1 immunostaining (green) was localized in the whole cytoplasm of the enterocytes under healthy and diseased conditions while the intensity of PDZK1 was markedly reduced in IL-10^−/−^ mice. NHE3 staining (red) was not reduced in IL-10^−/−^ mice. Nuclei of the cells were stained with DAPI (blue). A and B show representative images of three independent experiments. (C) Densitometric quantification of the NHE3, NHERF2, and PDZK1 protein expression in enterocytes of WT and IL-10^−/−^ mice. Densities are expressed in mean fluorescence intensities per pixel as means ± SEM. n = 4 animals; in each animal, 9–23 enterocytes in the same cell section plane were analyzed. *p<0.01 versus control.

To further investigate the functional NHE3 transport defect, we also studied the protein expression of NHE3 and the PDZ-adaptor proteins NHERF2 and PDZK1 by double immunofluorescence. NHE3 protein expression in the colonic mucosa of IL-10^−/−^ and WT mice was restricted to the apical membrane of the enterocytes without differences in localization and staining intensity (Figure 5A in green and Figure 5B in red). NHE3 immunostaining was not detected in other intestinal cell types in the epithelial layer of the mucosa. NHERF2 protein expression (Figure 5A in red) in the form of dotted areas, located in close vicinity to the NHE3 staining in the apical region of the enterocytes, was strongly reduced in the inflamed colon. PDZK1 staining in the enterocytes showed a more diffuse distribution in the cytoplasm, but the protein expression was also strongly reduced in the inflamed colon compared to the control samples (Figure 5B in green).

### Densitometric Quantification of NHE3, NHERF2, and PDZK1 Protein Expression

Changes in NHE3, NHERF2, and PDZK1 protein expression in the enterocytes of IL-10^−/−^ and WT mice were quantified by a computer-assisted densitometric method using microscopic fluorescence illumination (Figure 5C). NHE3 expression did not differ in IL-10^−/−^ mice compared to WT mice. NHERF2 expression was significantly reduced by 62% in the inflamed colon. PDZK1 expression in the enterocytes of the inflamed colon was also significantly reduced by 81% compared to the control samples.

### Fluorometric Measurements of NHE3 Transport Activity in Colonic Enterocytes

NHE3 mRNA and protein expression was not altered in the inflamed colon of IL-10^−/−^ mice and the brush border membrane localization was unaltered. The mRNA and protein expression of two of the NHE3 regulatory PDZ proteins of the NHERF family (PDZK1 and NHERF2) were downregulated in the inflamed colonic epithelium. Since PDZK1 reduction but not NHERF2 reduction has been shown to reduce acid-activated NHE3 activity, we analyzed NHE3 transport function in IL-10^−/−^ and WT colonic enterocytes. To assess the NHE3 transport activity, we measured acid-activated, Hoe642-insensitive, Na^+^-dependent proton flux in isolated colonic enterocytes (Figure 3A). The experimentally created transmembrane H^+^ and Na^+^ gradient was used as the driving force in this fluorometric measurement. In the presence of Hoe642, NHE1 and NHE2 were inhibited so that NHE3 almost completely mediated the acid-activated sodium-dependent proton flux. Under these experimental conditions, NHE3 activity was significantly reduced in the apical region of isolated colonic enterocytes from IL-10^−/−^ mice as compared to WT mice (Figure 3B).

### Comparison of the IL-10^−/−^ Mouse Model with the DSS Mouse, Another Model of IBD

To confirm the results in another mouse model of chronic colonic inflammation, we performed a comparison with the chronic DSS mouse model of IBD. In the colon of DSS treated mice H&E staining showed clear signs of inflammation characterized by an increase in mononuclear cells, infiltrating the mucosal and submucosal layer and some single cells migrating into the muscularis layer (H&E sections not shown). The mucosal layer in the inflamed colon analyzed in this chronic stage was intact but mildly hyperplastic. In the colon of non-treated control mice, mononuclear cells were only occasionally observed, mostly in the mucosal layer. A semiquantitative analysis yielded for the colon from control and DSS treated mice histopathological scores of 0 and 2.7±0.3, respectively (n = 3 mice in each group). Changes of mRNA expression profiles by *q*RT-PCR were comparable (Table S2). The expression of IL-1β and TNF-α as well as of iNOS, but not of IFN-γ and procaspase 3, were significantly increased. Gene expression of PDZK1 was also significantly decreased while that of NHE3 as well as of NHERF1 and NHERF2 were not changed (Table S2).

## Discussion

IBD in the IL-10^−/−^ mouse model was characterized by an immune cell infiltration comprising T-lymphocytes and macrophages in the mucosa and submucosa of the colon, confirming earlier observations [20], [21]. We show here that these immune cells were activated and produced proinflammatory cytokines, a combination of high levels of IL-1β and TNF-α, as documented at both the gene and protein expression levels by *q*RT-PCR, *in situ* RT-PCR, and immunohistochemistry. These parallel analyses of the gene and protein expression of proinflammatory cytokines allowed reliable identification of the immune cell activation status [22]–[24]. The results show that in contrast to the infiltrating immune cells, epithelial cells did not express the proinflammatory cytokines IL-1β and TNF-α, neither at the gene nor protein level. IFN-γ gene expression, originating from single activated infiltrating immune cells, was not increased in this chronic inflammatory state, confirming reports in other autoimmune diseases [22], [25]. IL-1β typically induces iNOS expression in activated infiltrating immune cells [26], as documented through expression of this inducible nitric oxide (NO) generating enzyme in the present study. In addition, very high expression levels of the proinflammatory cytokine TNF-α were detected at both the gene and protein expression levels in the immune cells. The additional presence of TNF-α potentiates the toxicity of IL-1β by fostering superoxide radical formation [27]. Treatment with anti–TNF-α antibody ameliorates mucosal inflammation and diarrhea in the IL-10^−/−^ mouse model [28], as well as in humans [8], [9], indicating that this proinflammatory cytokine is critical for the disease process; however, TNF-α antibody therapy does not suppress IL-1β effects. Thus, IL-1β–induced NO production alone, without concomitant superoxide radical formation resulting from TNF-α activity, is apparently not sufficient to cause intestinal dysfunction.

Many studies in humans and mice have shown that intestinal inflammation causes a functional disturbance of electroneutral sodium absorption [10]. NHE3 is the most important sodium absorptive transporter and responsible for the majority of electroneutral salt absorption in the intestine [11], and is the only functionally recognized sodium absorptive transporter in the proximal-mid colon, the area most affected by IL-10^−/−^ colitis [17]. Data on NHE3 expression in chronic intestinal inflammation are inconsistent, with both up- and downregulation as well as unchanged expression having been reported [29]–[33]. In addition, salt absorption in the intestinal mucosa may be disturbed because of a backflux of Na^+^ through leaky tight junctions, as demonstrated in a number of studies [34], [35]. In order to selectively study NHE activity in colonocytes, we therefore isolated colonic crypts and assessed fluorometrically acid-activated Na^+^/H^+^ exchange activity in the surface colonocytes in the mouths of the cryptal opening, which is >85% due to NHE3, as demonstrated before [18]. The present study shows that NHE3 transport activity in this mouse model of chronic intestinal inflammation was dysfunctional, as evidenced by reduced acid-activated NHE3 transport activity. This dysfunction was observed in spite of constitutive NHE3 gene expression as documented by *q*RT-PCR and for the first time by *in situ* RT-PCR, allowing a cell type specific identification of gene expression changes. Protein expression of NHE3 in the plasma membrane of the epithelial cells, as measured by immunofluorescence combined with quantitative densitometric analysis, was also not significantly affected without evidence for internalization of the protein from the membrane. Western blot analyses confirmed this observation (data not shown). This observation is in accordance with recent functional analyses in human colon biopsies of ulcerative colitis, which also revealed NHE3 dysfunction despite unchanged NHE3 expression [33].

So far, little is known about NHE3 transport regulation in the chronically inflamed intestine. PDZ-adaptors are scaffolding proteins, playing a central role in targeting, membrane retention, and signal complex formation of membrane proteins, including NHE3 [14]. Transgenic animal studies have shown that not only NHE3 deficiency [36] but also PDZ-adaptor protein deficiency as observed in NHERF1- [19], [37], NHERF2- [38], and PDZK1- [18], [39] knockout mice, can cause intestinal NHE3 dysfunction. To further investigate the NHE3 transport dysfunction in this animal model of chronic IBD, we therefore measured the expression of PDZ-adaptor proteins of the NHERF family. Indeed, we found that in IL-10^−/−^ mice, NHERF2 and PDZK1 expression at both the gene and protein levels were significantly decreased. In addition to using *in situ* RT-PCR and immunofluorescence to demonstrate downregulation of these two PDZ-adaptor proteins, we also quantified for the first time NHE3, NHERF2, and PDZK1 protein expression in enterocytes using computer-assisted densitometry. These are the first data which document a NHERF protein expression with cellular localization in the murine colonic epithelium.

How may the downregulation of these NHERF adaptors be related to the observed NHE3 dysfunction? NHERF2 deficiency in murine colon has been reported to selectively disturb certain regulatory features of NHE3, such as Ca^2+^-dependent NHE3 transport inhibition, without a change in acid-activated NHE3 transport [19]. In contrast, PDZK1 deficiency results in a strong decrease in acid-activated NHE3 activity in murine colonic surface cells [18]. The latter defect is very similar to the strong decrease in acid-activated NHE3 activity observed in the surface colonocytes of the IL-10–deficient chronically inflamed colon in this study, as well as the decreased acid-activated NHE3 activity observed in surface colonocytes of moderately inflamed mucosa from ulcerative colitis patients [33], [40]. We therefore assume that the very strong PDZK1 downregulation observed in IL-10^−/−^ colonocytes is a likely contributory factor to NHE3 dysfunction. This assumption will have to be confirmed in future experimentation.

We also compared the results obtained in the spontaneous IL-10^−/−^ mouse model with the situation in the DSS mouse, a chemically-induced IBD model. The histopathological scores were in the same range and the mucosal layer was preserved. The changes in the mRNA expression profile in the DSS mouse colon with significant increases of the proinflammatory cytokines IL-1β and TNF-α as well as a significantly decreased PDZK1 gene expression in spite of unchanged expression levels of NHE3 and NHERF1 were comparable to those in the IL-10^−/−^ mouse colon. The only significant difference between the two animal models was the lack of a reduction of NHERF2 gene expression in the DSS mouse colon. Severe colonic NHE3 dysfunction in DSS colitis has previously been described by our group [41]. Thus, NHE3 dysfunction despite normal NHE3 gene expression, but strongly reduced PDZK1 expression, is seen in both mouse models of chronic colonic inflammation. Data obtained in control experiments from IL-10^−/−^ mice kept under SPF conditions with non-inflamed colonic mucosa confirmed that there is no PDZK1 downregulation without inflammation.

Taken together, the present data support the idea of an interference of proinflammatory cytokines with NHE3 transport function despite unaltered NHE3 expression and membrane localization in the IL-10^−/−^ mouse model. Similar findings were obtained in the chronic DSS colitis mouse model. Downregulation of the PDZ-adaptor protein PDZK1 of the NHERF family was observed in both models as well. We therefore propose that NHE3 functional dysregulation may, at least in part, be related to NHE3 protein network disruption. Inflammation-induced PDZK1 downregulation may therefore be a contributory factor in the pathogenesis of inflammatory diarrhea in IBD.

## Materials and Methods

### IL-10–deficient Mouse Model

IL-10–deficient (*Il10^tm1Cgn^, Il10^−/−^*) and wild-type (WT) mice on a C57BL/6J background were produced and maintained in a room with a controlled environment (21±2°C, 55±5% relative humidity, 12 hours light/dark cycle). Mice were housed separated by sex in cages with a 360-cm^2^ floor area at a maximum of four animals on bedding of non-sterilized, dust-free softwood fibers. A pelleted diet (Altromin® 1314, Lage, Germany) containing 22.5% protein, 5.0% fat, and 4.5% fiber and tap water treated with UV light were provided *ad libitum*. Routine microbiological monitoring according to FELASA recommendations [42] did not reveal any evidence of infection with common murine pathogens except for *Pasteurella pneumotropica* and *Helicobacter hepaticus*. IL-10^−/−^ mice developed a disease that became apparent by weight loss and pasty stool at the age of 3–4 months. Mice were used at 8±1 months of age with a significant body weight reduction (24.1 g versus 28.4 g in WT mice, p<0.01). Additional IL-10–deficient mice were bred and maintained under strict specific pathogen-free (SPF) barrier conditions (IL-10^−/−^ SPF) as described previously [43]. IL-10^−/−^ SPF mice showed no clinical signs of inflammation with a body weight not significantly different to WT mice (27.5 g versus 28.4 g).

### DSS Mouse Model

12- to 14-weeks old male C57BL/6J mice (body weight: 21–22 g) were treated with 3% of dextran sodium sulfate (DSS; MW: 36,000–50,000 Da, MP Biomedicals, Eschwege, Germany) dissolved in autoclaved drinking water and subsequently sterilized by filtration. Drinking water was replaced by the DSS solution for five days followed by a 10-day recovery period. This treatment schedule was repeated for another three cycles. Body weight was monitored daily. Colitis manifested with typical clinical symptoms such as weight loss, diarrhea and rectal bleeding. Treatment caused a body weight loss of 2–3 g, which was normalized again towards the end of the treatment free intervals. Animal experiments were approved by the Local Institutional Animal Care and Research Advisory Committee at the Hannover Medical School and authorized by the local government for the regulation of animal welfare (Niedersächsisches Landesamt für Verbraucherschutz und Lebensmittelsicherheit).

### RNA Isolation, cDNA Preparation, and Real-time PCR

Segments of proximal colonic mucosa were dissected free of seromuscular layers. Total RNA was obtained using the NucleoSpin RNA/Protein kit (Machery & Nagel, Germany). RNA was reverse transcribed into complementary DNA using an oligo-dT18TNV primer (Fermentas, St. Leon-Rot, Germany) and the Revert-Aid™ H-Minus M-MuLV reverse transcriptase (Fermentas, St. Leon-Rot, Germany). Quantitative real-time reverse transcription polymerase chain reaction (*q*RT-PCR) was performed with the QuantiTect SYBR Green™ technology (QIAGEN, Hilden, Germany) using the DNA Engine Opticon™ Sequence Detection System (Biozym Diagnostik, Hessisch-Oldendorf, Germany). ß-actin was used for normalization. Table S3 shows the *q*RT-PCR primers used in this study.

### Fluorometric Analyses

Preparation of colonic crypts and pH measurements were performed as previously described [18], [19]. In brief, for the pH_i_ measurements colonic crypts where loaded with BCECF and mounted onto a heated stage of an inverted microscope (Zeiss Axiovert 200, Carl Zeiss AG, Jena, Germany). Crypts were acidified using an ammonium prepulse (40 mM NH_4_Cl isotonically replacing NaCl), then perfused with a Na^+^-free buffer (TMA^+^ isotonically replacing Na^+^), until pH_i_ reached its lowest value plateau. Subsequently, 50 μM Hoe 642 was added to the Na^+^-free buffer. After 2–3 min, the buffer was switched to Na^+^-containing buffer, supplemented with 50 μM HOE642 and forskolin, if appropriate. Cells were exposed to alternating 440 and 495 nm light from a monochromator (Visichrome, Visitron Systems, Puchheim, Germany) with a 515-nm DCXR dichroic mirror and a 535-nm barrier filter (Chroma Technology, Rockingham, VT, USA) in the emission pathway and images digitalized. Calibration of the 440/495 ratio was performed as described [44]. Regions of interest (ROIs) were selected in the apical and basal part of the crypts. NHE3 activity was measured by calculating the initial rate of pH_i_ recovery (dpH/dt) from acidosis after re-administration of Na^+^ (Figure 3B). For the fluorometric measurements, Hoe642 was kindly provided by Sanofi-Aventis (Frankfurt, Germany). Nigericin and 2′,7′-bis-(2-carboxyethyl)-5-(and-6)-carboxyfluorescein (BCECF) were purchased from Molecular Probes (Leiden, Netherlands).

### Morphology

H&E staining was used for general assessment of intestinal inflammation. Histopathological scores were performed as previously described [20] to grade the degree of colonic inflammation from grade 0 to 4 with grade 0: no changes from normal tissue (no signs of inflammation); grade 1: very low level of leukocyte infiltration; grade 2: low level of leukocyte infiltration; grade 3: high level of leukocyte infiltration involving the submucosa and thickening of the colon wall; grade 4: transmural leukocyte infiltration, loss of goblet cells, thickening of the colonic wall.

For immunohistochemistry, tissue specimens of mouse colon were rinsed with ice-cold phosphate-buffered saline (PBS) and fixed in 4% paraformaldehyde in 0.15 M PBS, pH 7.3. Fixed tissue was embedded in paraffin. Mouse colon sections from the experimental groups were immunostained by a fluorescence method as previously described [45]. For the double fluorescence immunostaining, we used the following primary antibodies: CD3-T-cells (hamster anti-mouse, MCA1413 Serotec, Düsseldorf, Germany), CD68-macrophages (MCA1947 rat anti-mouse, Serotec, Düsseldorf, Germany), IL-1β, TNF-α (rabbit polyclonal, R&D Systems, Wiesbaden-Nordenstadt, Germany), NHE3 (rabbit polyclonal, Alpha Diagnostics, San Antonio, TX, USA), PDZK1 (M-16, sc-27289, goat polyclonal anti-mouse, Santa Cruz Biotechnology, Santa Cruz, CA, USA) and NHERF1 (rabbit polyclonal, Ab5199) and NHERF2 (rabbit polyclonal, Ab2170) (kindly provided by Prof. Chris Yun, Emory University, Atlanta, GA, USA). The primary antibodies were detected using species-specific secondary antibodies conjugated to the green fluorescent Cy2 and the red fluorescent dye Cy3 (MoBiTec, Göttingen, Germany), and by a counterstaining with 4′,6-diamidino-2-phenylindole (DAPI) in the mounting medium (Vectashield Mounting Medium with DAPI, Vector Laboratories, Burlingame, CA, USA).

### Densitometric Measurements

In order to verify possible changes in the densities of NHE3, NHERF2 and PDZK1 protein expression in the enterocytes of the colonic mucosa was densitometrically quantified. A computer-assisted method (cell P software, Olympus) using fluorescence illumination (filters for carbocyanine dyes Cy2 and Cy3) was employed using the BX61 upright microscope (Olympus Optical, Hamburg, Germany). After separation for each channel the mean fluorescence intensities per pixel of the protein expression ranged between 8 and 85 for all epithelial cells (n = 4 in each group) after subtraction of background staining. The protein expression data were expressed from 9–23 epithelial cells in each group in comparable cell areas with a sectioned nucleus.

### *In situ* RT-PCR

Sections from the colonic mucosa of IL-10^−/−^ and WT mice were fixed on the same 3-Chamber SuperFrost Plus™ slides and subjected to *in situ* reverse transcription polymerase chain reaction (*in situ* RT-PCR) gene expression analysis using a two-step protocol with reverse transcription and PCR amplification on a specific thermal cycler (PTC-200 Twin Tower DNA Engine, MJ Research, Waltham, MA, USA) as described in detail before [22]. Table S4 lists the primer sequences.

### Statistical Analysis

Analyses of the *q*RT-PCR data and of the standard curve for the genes were performed using the Opticon Monitor v. 1.07 (MJ Research, Inc., Waltham, MA, USA). Statistical analyses were performed using the Prism analysis program (Graphpad, San Diego, CA, USA). Data were tested for significance using the unpaired Student’s *t*-test with p<0.05 as the limit of significance. All data are expressed as means ± SEM (standard error of the means).

## Supporting Information

Table S1 **Gene expression profile of the proinflammatory cytokines IL-1β, TNF-α, IFN-γ, iNOS, the cell death marker procaspase 3 and NHE3 and the PDZ-adaptor proteins NHERF1, NHERF2 and PDZK1 in the colonic mucosa of WT and IL-10^−/−^ SPF mice.**
(DOC)

Table S2 **Gene expression profile of the proinflammatory cytokines IL-1β, TNF-α, IFN-γ and iNOS and the cell death marker procaspase 3 and the transporter and adaptor proteins NHE3, PDZK1, NHERF1 and NHERF2 in the colonic mucosa of control and DSS treated mice.**
(DOC)

Table S3 ***q*** **RT-PCR primer sequences.**
(DOC)

Table S4 ***In situ*** **RT-PCR primer sequences.**
(DOC)

## References

[1] Podolsky DK (2002). Inflammatory bowel disease.. N Engl J Med.

[2] Field M (2003). Intestinal ion transport and the pathophysiology of diarrhea.. J Clin Invest.

[3] Rogler G, Andus T (1998). Cytokines in inflammatory bowel disease.. World J Surg.

[4] Sanchez-Munoz F, Dominguez-Lopez A, Yamamoto-Furusho JK (2008). Role of cytokines in inflammatory bowel disease.. World J Gastroenterol.

[5] Neurath MF, Fuss I, Pasparakis M, Alexopoulou L, Haralambous S (1997). Predominant pathogenic role of tumor necrosis factor in experimental colitis in mice.. Eur J Immunol.

[6] Kollias G, Douni E, Kassiotis G, Kontoyiannis D (1999). The function of tumour necrosis factor and receptors in models of multi-organ inflammation, rheumatoid arthritis, multiple sclerosis and inflammatory bowel disease.. Ann Rheum Dis.

[7] Reimund JM, Wittersheim C, Dumont S, Muller CD, Baumann R (1996). Mucosal inflammatory cytokine production by intestinal biopsies in patients with ulcerative colitis and Crohn’s disease.. J Clin Immunol.

[8] Hanauer SB, Feagan BG, Lichtenstein GR, Mayer LF, Schreiber S (2002). Maintenance infliximab for Crohn’s disease: the ACCENT I randomised trial.. Lancet.

[9] Rutgeerts P, Sandborn WJ, Feagan BG, Reinisch W, Olson A (2005). Infliximab for induction and maintenance therapy for ulcerative colitis.. N Engl J Med.

[10] Seidler U, Lenzen H, Cinar A, Tessema T, Bleich A (2006). Molecular mechanisms of disturbed electrolyte transport in intestinal inflammation.. Ann N Y Acad Sci.

[11] Schultheis PJ, Clarke LL, Meneton P, Miller ML, Soleimani M (1998). Renal and intestinal absorptive defects in mice lacking the NHE3 Na^+^/H^+^ exchanger.. Nat Genet.

[12] Zachos NC, Tse M, Donowitz M (2005). Molecular physiology of intestinal Na^+^/H^+^ exchange.. Annu Rev Physiol.

[13] Donowitz M, Li X (2007). Regulatory binding partners and complexes of NHE3.. Physiol Rev.

[14] Lamprecht G, Seidler U (2006). The emerging role of PDZ adapter proteins for regulation of intestinal ion transport.. Am J Physiol Gastrointest Liver Physiol.

[15] Seidler U, Singh A, Chen M, Cinar A, Bachmann O (2009). Knockout mouse models for intestinal electrolyte transporters and regulatory PDZ adaptors: new insights into cystic fibrosis, secretory diarrhoea and fructose-induced hypertension.. Exp Physiol.

[16] Shenolikar S, Voltz JW, Cunningham R, Weinman EJ (2004). Regulation of ion transport by the NHERF family of PDZ proteins.. Physiology (Bethesda).

[17] Kühn R, Löhler J, Rennick D, Rajewsky K, Müller W (1993). Interleukin-10-deficient mice develop chronic enterocolitis.. Cell.

[18] Cinar A, Chen M, Riederer B, Bachmann O, Wiemann M (2007). NHE3 inhibition by cAMP and Ca2^+^ is abolished in PDZ-domain protein PDZK1-deficient murine enterocytes..

[19] Broere N, Chen M, Cinar A, Singh AK, Hillesheim J (2009). Defective jejunal and colonic salt absorption and altered Na(+)/H (+) exchanger 3 (NHE3) activity in NHE regulatory factor 1 (NHERF1) adaptor protein-deficient mice.. Pflugers Arch.

[20] Berg DJ, Davidson N, Kühn R, Müller W, Menon S (1996). Enterocolitis and colon cancer in interleukin-10-deficient mice are associated with aberrant cytokine production and CD4(+) TH1-like responses.. J Clin Invest.

[21] Rennick DM, Fort MM, Davidson NJ (1997). Studies with IL-10^−/−^ mice: an overview.. J Leukoc Biol.

[22] Jörns A, Günther A, Hedrich HJ, Wedekind D, Tiedge M (2005). Immune cell infiltration, cytokine expression, and beta-cell apoptosis during the development of type 1 diabetes in the spontaneously diabetic LEW.1AR1/Ztm-*iddm* rat.. Diabetes.

[23] Bell EB, Westermann J (2008). CD4 memory T cells on trial: immunological memory without a memory T cell.. Trends Immunol.

[24] Uno S, Imagawa A, Okita K, Sayama K, Moriwaki M (2007). Macrophages and dendritic cells infiltrating islets with or without beta cells produce tumour necrosis factor-alpha in patients with recent-onset type 1 diabetes.. Diabetologia.

[25] Davidson NJ, Hudak SA, Lesley RE, Menon S, Leach MW (1998). IL-12, but not IFN-gamma, plays a major role in sustaining the chronic phase of colitis in IL-10-deficient mice.. J Immunol.

[26] Dinarello CA (2009). Immunological and inflammatory functions of the interleukin-1 family.. Annu Rev Immunol.

[27] Kolios G, Valatas V, Ward SG (2004). Nitric oxide in inflammatory bowel disease: a universal messenger in an unsolved puzzle.. Immunology.

[28] Gratz R, Becker S, Sokolowski N, Schumann M, Bass D (2002). Murine monoclonal anti-TNF antibody administration has a beneficial effect on inflammatory bowel disease that develops in IL-10 knockout mice.. Dig Dis Sci.

[29] Lohi H, Makela S, Pulkkinen K, Hoglund P, Karjalainen-Lindsberg ML (2002). Upregulation of CFTR expression but not SLC26A3 and SLC9A3 in ulcerative colitis.. Am J Physiol Gastrointest Liver Physiol.

[30] Yang H, Jiang W, Furth EE, Wen X, Katz JP (1998). Intestinal inflammation reduces expression of DRA, a transporter responsible for congenital chloride diarrhea.. Am J Physiol.

[31] Siddique I, Hasan F, Khan I (2009). Suppression of Na^+^/H^+^ exchanger isoform-3 in human inflammatory bowel disease: lack of reversal by 5′-aminosalicylate treatment.. Scand J Gastroenterol.

[32] Sullivan S, Alex P, Dassopoulos T, Zachos NC, Iacobuzio-Donahue C (2009). Downregulation of sodium transporters and NHERF proteins in IBD patients and mouse colitis models: potential contributors to IBD-associated diarrhea.. Inflamm Bowel Dis.

[33] Yeruva S, Farkas K, Hubricht J, Rode K, Riederer B (2009). Preserved Na(+)/H(+) exchanger isoform 3 expression and localization, but decreased NHE3 function indicate regulatory sodium transport defect in ulcerative colitis.. Inflamm Bowel Dis.

[34] Heller F, Florian P, Bojarski C, Richter J, Christ M (2005). Interleukin-13 is the key effector Th2 cytokine in ulcerative colitis that affects epithelial tight junctions, apoptosis, and cell restitution.. Gastroenterology.

[35] Mankertz J, Schulzke JD (2007). Altered permeability in inflammatory bowel disease: pathophysiology and clinical implications.. Curr Opin Gastroenterol.

[36] Gawenis LR, Stien X, Shull GE, Schultheis PJ, Woo AL (2002). Intestinal NaCl transport in NHE2 and NHE3 knockout mice.. Am J Physiol Gastrointest Liver Physiol.

[37] Murtazina R, Kovbasnjuk O, Zachos NC, Li X, Chen Y (2007). Tissue-specific regulation of sodium/proton exchanger isoform 3 activity in Na(+)/H(+) exchanger regulatory factor 1 (NHERF1) null mice. cAMP inhibition is differentially dependent on NHERF1 and exchange protein directly activated by cAMP in ileum versus proximal tubule.. J Biol Chem.

[38] Lin S, Yeruva S, He P, Singh AK, Zhang H (2010). Lysophosphatidic acid stimulates the intestinal brush border Na(+)/H(+) exchanger 3 and fluid absorption via LPA(5) and NHERF2.. Gastroenterology.

[39] Hillesheim J, Riederer B, Tuo B, Chen M, Manns M (2007). Down regulation of small intestinal ion transport in PDZK1- (CAP70/NHERF3) deficient mice.. Pflugers Arch.

[40] Farkas K, Yeruva S, Rakonczay Z, Ludolph L, Molnar T (2011). New therapeutic targets in ulcerative colitis: the importance of ion transporters in the human colon.. Inflamm Bowel Dis.

[41] Ukena SN, Singh A, Dringenberg U, Engelhardt R, Seidler U (2007). Probiotic Escherichia coli Nissle 1917 inhibits leaky gut by enhancing mucosal integrity.. PLoS One.

[42] Nicklas W, Baneux P, Boot R, Decelle T, Deeny AA (2002). Recommendations for the health monitoring of rodent and rabbit colonies in breeding and experimental units.. Lab Anim.

[43] de Buhr MF, Mahler M, Geffers R, Hansen W, Westendorf AM (2006). Cd14, Gbp1, and Pla2g2a: three major candidate genes for experimental IBD identified by combining QTL and microarray analyses.. Physiol Genomics.

[44] Bachmann O, Rossmann H, Berger UV, Colledge WH, Ratcliff R (2003). cAMP-mediated regulation of murine intestinal/pancreatic Na^+^/HCO3- cotransporter subtype pNBC1.. Am J Physiol Gastrointest Liver Physiol.

[45] Arndt T, Wedekind D, Weiss H, Tiedge M, Lenzen S (2009). Prevention of spontaneous immune-mediated diabetes development in the LEW.1AR1-*iddm* rat by selective CD8+ T cell transfer is associated with a cytokine shift in the pancreas-draining lymph nodes.. Diabetologia.

