# Supplementary material for: Downregulation of the NHE3-Binding PDZ-Adaptor Protein PDZK1 Expression during Cytokine-Induced Inflammation in Interleukin-10–Deficient Mice
Source: PLoS One. 2012 Jul 27;7(7):e40657. doi: 10.1371/journal.pone.0040657 (PMC3407152; doi:10.1371/journal.pone.0040657)
Supplement: Table S1 — Gene expression profile of the proinflammatory cytokines IL-1β, TNF-α, IFN-γ, iNOS, the cell death marker procaspase 3 and NHE3 and the PDZ-adaptor proteins NHERF1, NHERF2 and PDZK1 in the colonic mucosa of WT and IL-10−/− SPF mice. (DOC) [file pone.0040657.s001.doc]

**Supporting Information**

**Table S1.** Gene expression profile of the proinflammatory cytokines IL-1β, TNF-α, IFN-γ, iNOS, the cell death marker procaspase 3 and NHE3 and the PDZ-adaptor proteins NHERF1, NHERF2 and PDZK1 in the colonic mucosa of WT and IL-10^-/-^ SPF mice.

| **Gene** | **WT control** | **IL-10^-/-^ SPF** |
| --- | --- | --- |
| IL-1β | 3.4 ± 1.0 | 0.2 ± 0.1* |
| TNF-α | 1.3 ± 0.3 | 0.5 ± 0.1 |
| IFN-γ | 0.007 ± 0.0 | 0.001 ± 0.0 |
| iNOS | 4.7 ± 3.3 | 1.1 ± 0.6 |
| Procaspase 3 | 36.9 ± 12.6 | 29.3 ± 11.1 |
| NHE3 | 38.2 ± 7.7 | 35.1 ± 10.5 |
| NHERF1 | 129.8 ± 42.8 | 123.6 ± 21.6 |
| NHERF2 | 7.5 ± 0.7 | 7.4 ± 0.8 |
| PDZK1 | 12.5 ± 3.6 | 9.8 ± 5.3 |

Results are expressed as the mean normalized expression. mRNA was quantified in relation to β-actin. Data are mean values ± SEM (from 5–6 experiments in each group). *p<0.05 versus control.
