# Supplementary material for: Downregulation of the NHE3-Binding PDZ-Adaptor Protein PDZK1 Expression during Cytokine-Induced Inflammation in Interleukin-10–Deficient Mice
Source: PLoS One. 2012 Jul 27;7(7):e40657. doi: 10.1371/journal.pone.0040657 (PMC3407152; doi:10.1371/journal.pone.0040657)
Supplement: Table S2 — Gene expression profile of the proinflammatory cytokines IL-1β, TNF-α, IFN-γ and iNOS and the cell death marker procaspase 3 and the transporter and adaptor proteins NHE3, PDZK1, NHERF1 and NHERF2 in the colonic mucosa of control and DSS treated mice. (DOC) [file pone.0040657.s002.doc]

**Supporting Information**

**Table S2.** Gene expression profile of the proinflammatory cytokines IL-1β, TNF-α, IFN-γ and iNOS and the cell death marker procaspase 3 and the transporter and adaptor proteins NHE3, PDZK1, NHERF1 and NHERF2 in the colonic mucosa of control and DSS treated mice.

| **Gene** | **Control** | **DSS** | **Fold change  against control** |
| --- | --- | --- | --- |
| IL-1β | 0.1 ± 0.05 | 4.3 ± 1.4* | 30.4 |
| TNF-α | 2.5 ± 1.1 | 6.2 ± 0.9* | 2.4 |
| IFN-γ | 0.007 ± 0.004 | 0.0005 ± 0.0001 | 0.07 |
| iNOS | 2.0 ± 0.4 | 24.6 ± 4.7* | 12.1 |
| Procaspase 3 | 20.5 ± 3.4 | 18.1 ± 3.4 | 0.9 |
| NHE3 | 34.4 ± 9.6 | 31.3 ± 9.5 | 0.9 |
| NHERF1 | 127 ± 30 | 89.5 ± 16 | 0.7 |
| NHERF2 | 315 ± 27 | 327 ± 39 | 1.0 |
| PDZK1 | 17.2 ± 2.9 | 5.0 ± 1.8* | 0.3 |

Data are expressed as the mean normalized expression and fold change against controls (from 8 experiments in each group). Data are mean values ± SEM. *p<0.05 versus control.
