# Supplementary material for: Downregulation of the NHE3-Binding PDZ-Adaptor Protein PDZK1 Expression during Cytokine-Induced Inflammation in Interleukin-10–Deficient Mice
Source: PLoS One. 2012 Jul 27;7(7):e40657. doi: 10.1371/journal.pone.0040657 (PMC3407152; doi:10.1371/journal.pone.0040657)
Supplement: Table S3 — q RT-PCR primer sequences. (DOC) [file pone.0040657.s003.doc]

**Supporting Information**

**Table S3.** *q*RT-PCR primer sequences

| **Gene** |  | **5’-Primer sequence-3’** |
| --- | --- | --- |
| β-actin | FW | AGA GGG AAA TCG TGC GTG AC |
|  | RV | CAA TAG TGA TGA CCT GGC CGT |
| TNF-α | FW | CAT CTT CTC AAA ATT CGA GTG ACA A |
|  | RV | TGG GAG TAG ACA AGG TAC AAC CC |
| IL-1β | FW | CAA CCA ACA AGT GAT ATT CTC CAT G |
|  | RV | GAT CCA CAC TCT CCA GCT GCA |
| IFN-γ | FW | TCA AGT GGC ATA GAT GTG GA AGA A |
|  | RV | TGG CTC TGC AGG ATT TTC ATG |
| iNOS | FW | CAG CTG GGC TGT ACA AAC CTT |
|  | RV | CAT TGG AAG TGA AGC GTT TCG |
| Procaspase 3 | FW | CCT CAG AGA GAC ATT CT GGG CC |
|  | RV | GCT GCT CCT TTT GCT ATG ATC TTC C |
| NHE3 | FW | AGG CCA CCA ACT ATG AAG AG |
|  | RV | AGG GGA GAA CAC GGG ATT ATC |
| NHERF1 | FW | AGA TCT GCC TCC AGC GAT AC |
|  | RV | TTC ATT TTT CTT GCT CCA GTC C |
| NHERF2 | FW | TAG TCG ATC CTG AGA CTG ATG |
|  | RV | ATT GTC CTT CTC TGA GCC TG |
| PDZK1 | FW | TGA CGG TGT GGT GGA AAT G |
|  | RV | TGG CAG TAA AGA AGT GGA GAG |

Primers used for *q*RT-PCR. FW-forward (sense) primer; RV-reverse (antisense) primer. All amplicons were designed exon-spanning and were in a size ranging from 100–250 bp.
