# Supplementary material for: Downregulation of the NHE3-Binding PDZ-Adaptor Protein PDZK1 Expression during Cytokine-Induced Inflammation in Interleukin-10–Deficient Mice
Source: PLoS One. 2012 Jul 27;7(7):e40657. doi: 10.1371/journal.pone.0040657 (PMC3407152; doi:10.1371/journal.pone.0040657)
Supplement: Table S4 — In situ RT-PCR primer sequences. (DOC) [file pone.0040657.s004.doc]

**Supporting Information**

**Table S4***. In situ* RT-PCR primer sequences

| **Gene** |  | **5’-Primer sequence-3’** |
| --- | --- | --- |
| β-actin | FW | ACA GCT GAG AGG GAA ATC GT |
|  | RV | CTG CTT GCT GAT CCA CAT CT |
| IL-1β | FW | GAC GTT CCC ATT AGA CAG CTG CAC T |
|  | RV | CTT TTC CAT CTT CTT CTT TGG GTA TTG |
| TNF-α | FW | CTA CTG AAC TTC GGG GTG ATC GGT C |
|  | RV | CTG GTA TGA GAT AGC AAA TCG GCT |
| IFN-γ | FW | TGG AGG AAC TGG CAA AAG GAT GG |
|  | RV | TGG TGG ACC ACT CGG ATG AGC |
| iNOS | FW | CTT CAA CAC CAA GGT TGT CTG CAT |
|  | RV | ATG TCA TGA GCA AAG GCG CAG AAC |
| Procaspase 3 | FW | GAG CAC TGG AAT GTC ATC TCG CTC TG |
|  | RV | TAC AGG AAG TCA GCC TCC ACC GGT ATC |
| NHE3 | FW | TGT ATA TCG AGC CAT TGG TGT |
|  | RV | CTT CAA ATT CAG CTC ATG GAA |
| NHERF1 | FW | ATC TTG GAC CTC AAC ATC TCC |
|  | RV | GAG GGA AGA CTT CTT GTC TGG |
| NHERF2 | FW | GAA CTC TCA ACT GGG GCT TAC |
|  | RV | CAG TCT CAG GAT CGA CTA CCA |
| PDZK1 | FW | GCG GAT CCT TCG CCA CAG AA |
|  | RV | ATC CCA GGG CCC GGT TTC TT |

FW-forward (sense) primer; RV-reverse (antisense) primer. The size of the amplicons detected by the forward and reverse primers for *in situ* RT-PCR are between 150–600 bp because the procedure for development of *in situ* RT-PCR showed otherwise diffusion artefacts.
